# Development and process evaluation of an educational intervention for overdose prevention and naloxone distribution by general practice trainees

**DOI:** 10.1186/s12909-015-0487-y

**Published:** 2015-11-20

**Authors:** Jan Klimas, Mairead Egan, Helen Tobin, Neil Coleman, Gerard Bury

**Affiliations:** 1 Centre for Emergency Medical Science, School of Medicine and Medical Science, University College Dublin, Dublin, Ireland; 2 British Columbia Centre for Excellence in HIV/AIDS, St. Paul’s Hospital, 608-1081 Burrard Street, Vancouver, British Columbia V6Z 1Y6 Canada; 3 c/o Coombe Family Practice, Dolphins barn, Dublin, Ireland

**Keywords:** Overdose, Feasibility study, Naloxone, Heroin, Education, General practice

## Abstract

**Background:**

Overdose is the most common cause of fatalities among opioid users. Naloxone is a life-saving medication for reversing opioid overdose. In Ireland, it is currently available to ambulance and emergency care services, but General Practitioners (GP) are in regular contact with opioid users and their families. This positions them to provide naloxone themselves or to instruct patients how to use it. The new Clinical Practice Guidelines of the Pre-hospital Emergency Care Council of Ireland allows trained bystanders to administer intranasal naloxone.

We describe the development and process evaluation of an educational intervention, designed to help GP trainees identify and manage opioid overdose with intranasal naloxone.

**Methods:**

Participants (*N* = 23) from one postgraduate training scheme in Ireland participated in a one-hour training session. The repeated-measures design, using the validated Opioid Overdose Knowledge (OOKS) and Attitudes (OOAS) Scales, examined changes immediately after training. Acceptability and satisfaction with training were measured with a self-administered questionnaire.

**Results:**

Knowledge of the risks of overdose and appropriate actions to be taken increased significantly post-training [OOKS mean difference, 3.52 (standard deviation 4.45); *P* < 0.001]; attitudes improved too [OOAS mean difference, 11.13 (SD 6.38); *P* < 0.001]. The most and least useful delivery methods were simulation and video, respectively.

**Conclusion:**

Appropriate training is a key requirement for the distribution of naloxone through general practice. In future studies, the knowledge from this pilot will be used to inform a train-the-trainer model, whereby healthcare professionals and other front-line service providers will be trained to instruct opioid users and their families in overdose prevention and naloxone use.

## Background

Overdose is the most common cause of death among people with opioid disorders and its prevention and management are thus priorities for healthcare agencies [1]. Europe has on average 17 drug-related deaths per million people (15–64 years) per year, varying from country to country [2]. With 70 drug-related deaths per million, Ireland has the third highest rate in Europe [2, 3]. Ambulance services in Dublin attend to an opioid overdose every day [4]. The use of the opioid antagonist naloxone is well recognised as an effective treatment for opioid overdose, and constitutes standard medical treatment in such situations. However, to prevent death, naloxone must be given very soon after the opioid has caused respiratory depression or arrest [5]. To date, naloxone has generally been used in injectable form, delivered via intramuscular, intravenous or intraosseous routes. A number of interventions to introduce naloxone to families, friends and drug workers have been established in countries other than Ireland and report positive effects [1, 6, 7], such as reduced death rates, less risk of needle stick injury or increased opioid overdose-related knowledge and competence. Ireland currently has no layperson distribution of naloxone; however, the government recently announced a lay demonstration project [8] which commenced in May 2015. Under this project, a total of 600 people with opioid use disorders will receive a multi-dose naloxone injector, a product with up to five injectable doses, to take home. Given the prevalence of blood-borne infections among the target group, the recent upsurge in HIV infections and many years of strenuous efforts to minimise injecting risks, the pitfalls of distributing a multi-dose injector have to be assessed. The feasibility of expanding the distribution of less risky intranasal naloxone via other channels such as primary care has yet to be determined [9, 10].

Bystanders, specifically frontline service providers, peers or family members of opioid users, are best positioned to intervene immediately when symptoms of overdose first appear [11]. General Practitioners (GP) in Ireland are also in regular contact with opioid users and their families, either via methadone maintenance treatment or other medical services in general practice. This access should allow GPs to provide naloxone themselves, or to instruct patients or family members on how to use it. However, no structured provision of naloxone exists in Irish general practice, and previous research elsewhere has shown that GPs lack skills and knowledge regarding naloxone administration and require more training [12]. Our preliminary work suggested that this training should include elements of the ‘Clinical Practice Guidelines (CPG) approved by the Pre-Hospital Emergency Care Council of Ireland in October 2013 (Emergency First Response)’, specifically initiating contact with emergency services, cardio-pulmonary resuscitation (CPR), and the administration of intranasal naloxone (INN) [13, 14]. However, the feasibility and acceptability of such training for GPs has not been previously reported. Therefore, the current study aimed to:

- Develop an educational intervention that enables doctors in specialist training for general practice to support bystander response to overdose (i.e., initiating contact with emergency services, CPR, etc.), and the administration of intranasal naloxone (INN).
- Determine the potential feasibility, acceptability and usefulness of this training to junGP trainees.
- Describe the process of development and evaluation.

## Methods

### Design, sample and intervention

Ireland’s population of 4.6 m is served by approximately 2,600 GPs; around 160 doctors enter one of 14 specialist-training programmes in GP each year. Each programme is accredited nationally and follows a standard four-year programme, the final two years of which are spent in supervised training practices. 23 GP trainees from the Dublin Mid Leinster Specialist Training Programme in general practice, affiliated with University College Dublin, were invited to participate in a one-hour training session. All accepted and took part in the study voluntarily. At the time, they were based in designated training general practices under the supervision of an accredited GP trainer.

Most practices were in Dublin (43 %), with 1000 or more patients on their General Medical Services list – this is a government subsidised health plan providing free point of care primary care and medicines for those on low incomes. Only six practices (26 %) prescribed methadone.

Most practices had one to three full-time GPs (16, 70 %) and one to four part-time GPs (15, 83 %). More than half of the practices had a practice nurse. Most trainees (91 %) ranged from 25–34 years old, and most were female (78 %). Eight (35 %) were trained methadone prescribers and 11 (48 %) had witnessed an opioid overdose (Table 1).

**Table 1:** Sample characteristics

	Number	Percent
Profiles of training practices		
County of practice		
Dublin	10	43 %
Wicklow	8	35 %
Other	3	12 %
Missing data	2	10 %
GMS list size		
<500	1	4 %
500–1000	4	17 %
1000–1500	7	30 %
1500–2000	2	9 %
>2000	8	35 %
Missing data	1	5 %
Practice setting		
Urban	9	39 %
Rural	5	22 %
Mixed	8	35 %
Missing data	1	5 %
Mean number of GPs (excluding GP registrars)		
Full time	2.6	(SD 2.0)
Part time	1.6	(SD 1.2)
Practice nurse	12	52 %
Active member of a Primary Care Team	11	48 %
Ever attended a primary care team meeting	6	26 %
Methadone prescribing	6	26 %
Level of methadone prescribing		
Level 1	4	17 %
Level 2	2	9 %
N of patients receiving methadone in the practice		
0–5	1	4 %
5–10	1	4 %
10–15	2	9 %
15–20	1	4 %
Years prescribing methadone		
3 years	1	4 %
15+ years	2	8 %
Trainee profile		
Age		
25–34 years	21	91 %
35+ years	2	9 %
Year of Graduation		
2008	5	22 %
2009	5	22 %
2010	4	17 %
2011	5	22 %
Other	3	12 %
Training in addiction		
0 h	1	4 %
<4 h	3	13 %
4–10 h	7	30 %
11–40 h	2	9 %
>40 h	1	4 %
Trained in methadone prescribing		
Level 1	8	35 %
None/Planned during training	15	65 %
(i) Ever witnessed an opioid overdose:		
Hospital	9	39 %
Community	2	9 %
(ii) Total No of Witnessed Hospital overdoses	39^a^+	
(iii) Total No of Witnessed Community overdoses	2	
(iv) Ever administered Naloxone outside of Emergency Department	5	22 %
Knowledge on Drugs in Ireland – Multiple-choice questions		
No of trainees who know how many people die due to overdose every year in Ireland	10^b^	43 %
No of trainees who know how many people are currently in methadone treatment in Ireland	8^c^	35 %

^a^One trainee witnessed more than 10 hospital overdoses

^b^Multiple-choice question options: (i) < 100___(ii)200-300___(iii) > 300___

^c^Multiple-choice question options: (i) < 3000___(ii)3000-6000___ (iii) > 6000___

### Ethical considerations/adherence to the International guidelines

The Irish College of General Practitioners Research Ethics Committee approved this study on August 27^th^, 2014. The research on human participants carried out in this study is in compliance with the Helsinki Declaration (http://www.wma.net/en/30publications/10policies/b3/index.html). This study adheres to the RATS guidelines on qualitative research (http://www.biomedcentral.com/ifora/rats). We informed the trainees about the study and gained their consent one week before the educational session. Our convenience sample is likely to be unrepresentative of the national profile of doctors in specialist training for GP.

### Development of the educational session

The educational session was developed as part of an evolving system of lay delivered INN. The key components of the system include:

1. A one-year prospective audit of the characteristics of opioid overdoses reported to ambulance services in Dublin [4, 15].
2. The development and implementation of CPG-led administration of naloxone [16].
3. The exploration of mechanisms for roll-out of naloxone by registered Medical Practitioners, since it remains a prescription-only drug in Ireland.

As a first step, a national Naloxone Advisory Group was established. A literature review on care options then determined the intervention of choice, i.e. the intranasal formulation. While it appears to address safety, efficacy and utility criteria, INN has not yet been approved by the Irish Department of Health for general use; intramuscular naloxone is currently available for prescription by doctors. However, an INN formulation is likely to become available in coming months, and INN-oriented training was identified as a long-term goal of the initiative. Our subsequent steps followed the Medical Research Council’s (MRC) framework, which advocates core phases in the development of health services interventions: preclinical, theoretical, modelling, exploratory trial, definitive trial and long-term implementation [17]. While the term preclinical usually refers to testing of interventions or medications in non-humans, the MRC framework defines its goal as: “Identifying the existing evidence and any theoretical basis for the intervention in order to describe the components of the intervention” (www.mrc.ac.uk/complex_packages.html).

In the preclinical stage of the intervention development, we identified a need and targets for naloxone distribution by geo-locating urban overdose hotspots in Dublin city – areas with high rates of overdoses [4]. They helped us to concentrate our efforts on general and addiction care services in inner city Dublin. Our participants were on training placements in practices neighboring the inner city hotspots. The subsequent modelling phase formulated clinical practice guidelines (CPG). The Pre-hospital Emergency Care Council (PHECC, the Statutory Regulator for Pre-Hospital Emergency Care in Ireland) approved these guidelines in October 2013. The UCD Centre for Emergency Medical Science concurrently collaborated with PHECC and the Naloxone Advisory Group to develop and pilot an educational session led by the guidelines. The guidelines allow for the training of lay people and health professionals in overdose prevention and naloxone use, subject to previous CPR training.

The PHECC Clinical Practice Guidelines recommend that naloxone training be provided as part of an overall emergency care package that includes Basic Life Support (BLS) skills training. There are two BLS levels prescribed by PHECC:

- Cardiac First Response - Community (CFR).
- Cardiac First Response - Advanced (CFR-A).

All trainees were required to achieve the CFR standard as a prerequisite of the session - this was already held by all participants. After the completion of this pilot study, the session will be evaluated by a group of community health professionals. Data from this feasibility evaluation will inform the design of the final stage of developing a national implementation for INN distribution.

### Content and delivery of the educational session

We based the intervention on our previous work, on pre-implementation assessments from Scotland and on training of family members to manage opioid overdose and administer naloxone in England [6]. More specifically, factors enabling naloxone distribution and use were incorporated into the educational session: evidence of effectiveness, appropriate training, and the development of a policy regulation – the CPG – that would allow intranasal administration [12, 18]. The intervention was facilitated by:

- a small group session.
- a practical exercise.
- a video clip using content from: a) the family work described in the above English study, b) the introduction of take-home IN naloxone within the National Health Service (NHS) Highland area [18].
- anonymous evaluation/feedback.

The video clip ensured the fidelity and consistency of the information distribution. This is an evidence-based methodology in emergency care training, and is used by emergency services globally [19]. Multi-media theory was reinforced at each stage with practical application and exercises. The video was three minutes in duration and its headings included:

- Recognition of overdose.
- Assembly of the drug administration system.
- INN administration.

The educational session was delivered by two facilitators in a group setting, and lasted approximately 45 min. It was held in the UCD Medical School. A manual for the trainers was developed before the delivery of the session in collaboration with the Naloxone Advisory Group, formed in the pre-clinical stages (http://drugs.ie/features/feature/naloxone_the_welsh_experience).

Our approach was informed by the experiential learning theory that allowed trainees to experience the process of Naloxone administration. Similar experiential curricula have improved addiction management skills and knowledge in medical students and paediatric residents elsewhere [20, 21]. The aims of the educational session, described in the current study, were to ensure that GP trainees had the skills to manage an overdose, i.e. initiating contact with emergency services, performing CPR, using INN and acquiring sufficient knowledge, understanding and motivation to be willing to undertake INN distribution and training. The key learning outcomes of the educational session were to teach GPs how to i) recognise opioid overdose, ii) assemble INN, and iii) administer INN (Table 2). Specific teaching and dissemination strategies for those receiving naloxone kits will depend on the recommendations of the current pilot project about whom should receive these kits (e.g. people who use drugs, family members, lay health/social care workers). Teaching and dissemination strategies will then be developed and included for recipient groups.

**Table 2:** Learning outcomes, delivery method/content and initial evaluation of the session

Learning outcomes
• To recognise opioid overdose • To assemble naloxone • To administer INN
Delivery method
• Formal presentation • Video demonstrations of how to i) recognise opioid overdose, ii) assemble naloxone, and iii) administer INN • Practical exercises on how to assemble and administer INN • Q & A discussion • Repeated measures assessment/feedback
Evaluation of education session
• Perceived changes in knowledge and attitudes • Qualitative data on strengths/weaknesses • Anonymous and confidential

### Data collection

A repeated-measures design using the validated Opioid Overdose Knowledge (OOKS) and Attitudes (OOAS) Scales examined changes immediately after the training session [22]. The acceptability of and satisfaction with this training were measured with a self-administered questionnaire immediately after the session, which included the acceptability of the session, learning needs and suggested improvements.

OOKS has 45 items organised in four sub-scales (risks, signs, actions and naloxone use, range 0–45). The OOAS has 28 items grouped in three sub-scales (competence, concerns and readiness, range 28–140). Both scales were developed and psychometrically evaluated with a convenience sample of friends and family members of heroin users and healthcare professionals in England. Both OOKS and OOAS were shown to be internally reliable (Cronbach’s alpha = 0.83 and 0.90, respectively). Retest after 14 days also showed fair-to-excellent values (OOKS, ICC = 0.90 and OOAS, ICC = 0.82). Professionals scored significantly higher on both scales than family members [22]. We changed two questions about “needles” and deleted two items about “injecting” naloxone in the attitudes scale (new range 26–130). The original scales were for injectable Naloxone; we used the intranasal formulation. We acknowledge the potential threat to validity and reliability, although the changes were minor.

The *acceptability* of the single session to trainees was assessed with open-ended questions that asked them to write what was good or bad about each of the five training delivery methods, or teaching modalities. The trainees rated each session based on its usefulness (5-point Likert scales); the rating scales were taken from our previous study [23].

### Data analysis

We used the non-parametric Wilcoxon Paired Signed Rank test for our analysis (IBM SPSS, version 20). We report means and standard deviations. For the composite usefulness score, all participants’ Likert scale scores were added together, and the means and standard deviations (SD) calculated. Answers to open-ended questions underwent content analysis using the questions as the codes; similar responses were grouped, groups were titled and the number of responses counted. Using conventional content analysis, coding groups (categories) were derived directly from the text data [24].

## Results

### Pre-training and post-training knowledge

The educational session elicited significant changes in three out of four knowledge categories (i.e., risks, actions and use of naloxone, see Table 3). Furthermore, the median composite knowledge score increased from 28 pre-training to 31 post-training (*p* < 0.001).

**Table 3:** Self-reported change in knowledge and attitudes pre-/post-training, and usefulness of the session

Knowledge/attitudes	Pre-training median/mean (SD)	Post-training median/mean (SD)	Mean diff (SD)	Wilcoxon Z/*P*-value
Knowledge:	28/27.9 (4.5)	31/31.4 (1.5)	3.5 (4.5)	−3.50, 0.000
Risks	8/7.5 (1.9)	9/8.7 (0.7)	1.17 (2.1)	−2.69, 0.007
Signs	6/6.0 (1.8)	6/6.4 (0.7)	0.4 (1.9)	−0.80, 0.422
Actions	6/5.5 (1.2)	6/6.4 (1.1)	1.0 (1.3)	−3.04, 0.002
INN use	9/9.0 (1.2)	10/10.0 (0.7)	1.1 (1.3)	−3.09, 0.002
Attitudes:	96/97.4 (7.2)	108/108.6 (8.1)	11.1 (6.4)	−4.11, 0.000
Competencies	33/33.7 (4.7)	41/41.0 (3.9)	7.4 (5.0)	−4.11, 0.000
Concerns	22/22.1 (2.6)	24/24.0 (2.9)	2.0 (2.2)	−3.46, 0.001
Readiness	40/41.7 (3.3)	43/43.5 (3.9)	1.7 (2.8)	−2.63, 0.008
The following were useful in education	Completely agree/agree N (%)	Unsure	Completely disagree/disagree N (%)	Mean score post-training (SD)
Presentation	23 (100)	0	0	4.5 (0.5)
Video	19 (82.6)	3 (13.0)	1 (4.3)	4.2 (0.8)
Simulation	23 (100)	0	0	4.6 (0.5)
Q & A discussion	21 (91.3)	2 (8.7)	0	4.3 (0.6)
Guideline demonstration	20 (87)	3 (13.0)	0	4.4 (0.7)

### Skills

All participants were directly observed to have acquired the skills needed to assemble and effectively deliver the correct dose of naloxone in a safe manner. All delivered INN following the procedures described by the CPG, rather than simply spraying the dose into the nose.

### Pre-training and post-training attitudes

There was a significant increase in all three categories (competencies, concerns and readiness) of positive attitudes towards overdose management (Table 3). The median composite score for attitudes increased from 96 pre-training to 108 post-training (*p* < 0.001).

### Evaluation of the educational session

The group mean for the session usefulness score was 21.9 (out of 25); the most and the least useful delivery methods were simulation and video respectively (see Table 4).

**Table 4:** Acceptability of the educational session

How did you find each aspect of the session?		
	What was good about it?	How can it be improved?
Presentation	- Clear 4/15^a^ - Informative 7/15 - Concise 8/15	- Less rushed, more interactive 2/3 - Stimulating questions 1/3
Video	- Visual 3/11 - Practical or demonstrative 5/11 - Clear 2/11	- Audio 6/7 - More time 1/7
Simulation	- Hands on experience of usage 13/18 - Informative 2/18 - Demonstrated ease of use, increased confidence 3/18	- More time 2/6 - Practice 1/6 - Facilitators 1/6 - Sound 1/6
Q & A discussion	- Opportunity to ask questions 4/6 - Collaborative 1/6 - Good/clear 2/6	- No major questions asked 2/5 - More time 1/5 - Naloxone for lay people and access for GPs 1/5
Would any other educational interventions/activities help trainees?		
• Booster sessions 1/9 • More simulations/real life situations 3/9 • More samples, syringes, differences between IN and exact-dose-dispenser 4/9		
Suggestions for improvement:		
• Booster sessions 3/8 • More time 2/8 • Scenarios 1/8 • Very/good 2/8		

^a^Fractions indicate how many trainees reported about the particular item out of the total number of trainees who responded to the question

Most of the participants (74 %) felt their questions were answered and saw a potential for the INN or overdose prevention in their training practice. The part of the presentation that trainees liked the most was that it “*provided answers to the questions I had just asked”* (participant quote). It could have been *“less rushed, more interactive”* (participant quote).

In the video, the trainees were able to “*actually see the device* [Mucosal Atomiser Device]” (participant quote). The video’s sound could be improved. During the practical simulation it was “*helpful to see how easy it is* [administration]” (participant quote); more time could be spent on this. The trainees perceived the small Q & A discussion as an “*opportunity to ask questions”* (participant quote). One commented, “*would be nice to discuss pros/cons of lay people having naloxone and where GP would avail of it*”.

Finally, trainees were given an opportunity to comment on their educational needs or provide suggestions for improving the session (Table 4). Several wanted more examples or real life situations to try, and two other trainees wished for more time or booster sessions: “*very quick session so difficult to fully answer all Q’s* [questions]*, however, very useful and would definitely allow us/help us to know what to do in OD setting”* (participant quote).

## Discussion

This educational session, informed by a Clinical Practice Guideline (CPG), has significantly improved knowledge of and positive attitudes towards overdose management among GP trainees. Based on the mean Likert-scales scores, the most useful components of the training were simulation, presentation and Q & A discussion, with GP trainees appreciating the opportunity to ask questions.

Our findings are consistent with the literature that highlights the effectiveness of education in improving knowledge of and attitudes towards overdose management [25, 26]. Other studies successfully trained people who used drugs [27], their families or friends [6], needle exchange workers [28], police and fire-fighters [29]. The various lengths and formats of training reported in this literature suggest that less training may be needed than we thought [30]; for instance, participants in a recent UK trial saved a comparable number of people with naloxone regardless of whether they received the full training or information only (five vs three controls), over a three months follow up [6]. Our training session produced slightly higher changes in positive attitudes, and compared to the UK trial it was linked with greater competence and confidence, though we could not demonstrate impact on the provider behaviour in an overdose situation. The changes in the attitudes towards and willingness to intervene in an opioid overdose suggest that our trainees would have used naloxone should they be provided with a take-home dose.

The feasibility and acceptability of our session for GP trainees were comparable with previous research in other groups [29, 31–33]. In this study, some aspects of the educational session were more helpful than has been reported in previous literature, i.e. hands-on experience with materials and GP access to INN kit [27].

The focus of the training session developed in this pilot project was on intranasal naloxone and general practice (GP). This hasn’t been done before. While the target population of the training session was unusual, the GP trainees clearly demonstrated improved skill, knowledge and willingness to intervene in a possible opioid overdose. Recognising this implication should shift our thinking about the role of GPs in the management and prevention of overdoses. In the literature, GPs tend to be overlooked as a possible training or distribution avenue. This route may be a unique component of a national roll-out of the naloxone strategy [34], and, as evident in our findings, one acceptable to doctors in training. The decision to focus the educational session on the GP trainees was influenced mainly by a recent Scottish pre-implementation study [12], and the frequent contact that GPs have with patients in methadone maintenance treatment in Ireland [35] or elsewhere [36]. The Scottish pre-implementation study indicated that general practice may be a viable route for distributing naloxone in the community; while half of the GPs were unsure about GP-based naloxone, the other half were willing to provide this drug to family or buddies of opioid users.

Intranasal naloxone (INN) is a needleless, safe and effective alternative to intramuscular formulations [13, 14, 37–39]. Future studies should use INN for training and distribution, especially because of its safety for both bystanders (e.g. reduced fear of injury), and for opioid users (e.g. less suspicion from police if naloxone is found, although in the United States, it does not mean less suspicion or problems with the police and people may fear that possession of naloxone means that the person is using drugs.). The legal situation in Ireland is currently under governmental review and changes in the legal status of Naloxone are expected to make it more broadly available [40]. If the current prescription-only status is relaxed, GPs may have greater clarity about their prescribing responsibilities and more flexibility in their dissemination of these kits. The challenge for future research and education is also to incorporate INN training into medical education and to engage other groups of service providers and clients to use INN and to prevent overdoses.

The current study is limited in several ways. Our findings are not generalizable to the larger population of GPs involved in addiction treatment. The GP trainees participated voluntarily, and were not obliged to take part in the training or to apply their learning in practice. Just because doctors can use naloxone does not imply that they can or will train others in its use. It would be useful to include qualitative questions about provider intent to use their new knowledge. Our core focus on the application of a validated Framework For Development Of Complex Health Interventions by U.K. Medical Research Council (MRC) [17], together with the repeated-measures design, suggests the intervention’s potential, and future research may determine whether it is generalisable to other GPs inside or outside of Ireland. For example, because providers in different locations were not assessed, it would be worthwhile to pursue this in a future study. The results from this study can be used to tailor the training session for physicians and re-evaluate it. It would be worthwhile to do longer-term follow up of this group to see if they retained their knowledge or positive attitudes, changed their practice, or both. Given the previous trial among British families [6], a randomized evaluation of the INN would be worthwhile.

## Conclusion

General practice trainees can be trained to support bystander response to overdose with intranasal naloxone. Appropriate training is a key requirement for the distribution of naloxone through general practice. In future studies, our educational session should be used to inform a train-the-trainer model, whereby GPs and healthcare professionals or other frontline service providers are trained to train opioid users and their families in overdose prevention and naloxone use. If feasible, such research can expand the role of general practice in the management of opioid overdose and the distribution of naloxone to opioid users, friends, families, frontline service providers and other professions.

## Availability of supporting data

None.

## Abbreviations

BLS: Basic Life Support
CFR: Cardiac First Response - Community (CFR)
CFR-A: Cardiac First Response - Advanced (CFR-A)
CPG: Clinical Practice Guidelines
CPR: cardio-pulmonary resuscitation
GP: General Practice
ICC: intracluster correlation coefficient
INN: intranasal naloxone
MRC: Medical Research Council
NHS: National Health Service
OOAS: opioid overdose attitudes scale
OOKS: opioid overdose knowledge scale
PHECC: The Pre-hospital Emergency Care Council of Ireland
SD: standard deviation
UCD: University College Dublin
